# Nutrition for Prevention and Control of Chronic Degenerative Diseases and COVID-19

**DOI:** 10.3390/nu15102253

**Published:** 2023-05-10

**Authors:** Laura Di Renzo, Paola Gualtieri, Giulia Frank, Antonino De Lorenzo

**Affiliations:** 1 Section of Clinical Nutrition and Nutrigenomic, Department of Biomedicine and Prevention, University of Tor Vergata, Via Montpellier 1, 00133 Rome, Italy; laura.di.renzo@uniroma2.it (L.D.R.); delorenzo@uniroma2.it (A.D.L.); 2 School of Applied Medical-Surgical Sciences, University of Tor Vergata, Via Montpellier 1, 00133 Rome, Italy; giulia.frank@ymail.com

Chronic degenerative diseases (CDD) are non-infectious, slow-progressing, and long-lasting diseases such as diabetes, heart disease, obesity, chronic respiratory diseases, neurodegenerative diseases, and cancer. CDDs have thus far been the most frequent causes of long-term disability and death worldwide. Globally, more than 30% of the population is affected by one or more CDDs, and 70% of public health resources are used for these diseases. Meanwhile, a real and current global threat is infectious diseases caused by emerging pathogens, such as SARS coronavirus 2 (SARS-CoV-2). The relationship between CCD and susceptibility to viral infections cannot be denied at this particular time. Recent findings show that the most severe clinical conditions of COVID-19 have been observed in persons with metabolic dysfunction and obesity, as well as those with micronutrient deficiencies and protein–energy malnutrition.

Yokoro et al. [1] highlighted the relationship between elderly frailty and diet quality during the COVID-19 pandemic. According to the survey results, the elderly lost the motivation to follow a healthy diet and about 10% of the respondents developed frailty. A significant association was observed between the dietary variety score and the frailty score after adjustment for sex and age, smoking, alcohol use, body mass index (BMI), and living alone. Therefore, in the elderly, to reduce the risk of disease due to reduced dietary variety, dietary supplementation may be necessary.

Susceptibility to viral infections, including SARS-CoV-2, depends on immune system dysfunction and the subsequent loss of homeostatic balance between regulatory T cells (TREG), which act to suppress immune response, and T helper 17 cells (Th17), characterized by the production of inflammatory cytokines and interleukin-17.

High dietary inflammatory index (DII)/energy-adjusted DII (E-DII) scores increased COVID-19 risk by 10 to 17%, and the highest DII quintile was observed to increase COVID-19-related death risk by 43% [2].

As malnutrition impairs immune function, enriched feeding with certain trace elements and antioxidant compounds, vitamins, and minerals could be useful to modulate immune and inflammatory responses and improve patient outcomes. Vitamin D (cholecalciferol), vitamin C (ascorbic acid), and zinc (Zn; zinc sulfate monohydrate) were significantly associated with a reduced risk of COVID-19 infection and severity [3]. Additionally, the timing of antioxidant intake was significantly associated with reduced hospitalization [3], and vitamin C, a powerful antioxidant, was found to reduce the length of stay in the intensive care unit (ICU) and hospital mortality [4]. A significant reduction in hospital mortality of 39.3% vs. 51.8% was observed with and without vitamin C supplementation, respectively, in randomized controlled trials conducted in China, Iran, Turkey, the USA, Pakistan, Saudi Arabia, and Greece [4]. Furthermore, supplementation with vitamins of the B group, vitamin C, and vitamin D could reduce low-grade inflammation observed in patients affected by chronic kidney disease (CKD), periodontal diseases, and dysbiosis [5].

Magnesium, as the cofactor in vitamin D metabolism, exerted immunoregulatory activity with beneficial effects on erythrocyte homeostasis [6].

Tian J. et al. highlighted that low serum magnesium concentrations were negatively correlated with the severity of COVID-19 [6]. By recombining surveillance data of COVID-19 cases from the US Centers for Disease Control and Prevention (CDC) with county-level ambient magnesium concentration data from the National Geochemical Survey (NGS), a large retrospective cohort study conducted in the USA observed a higher cumulative incidence of COVID-19 at the county level in low-magnesium areas.

Supplementation with essential trace elements, such as iron (Fe), Zn, copper (Cu), and selenium (Se), plays a key role in the prevention of CCDs, such as type-2 diabetes mellitus, and in the pathogenesis and prevention of chronic hepatitis B, C, and E, as well as coronavirus-19, by deregulating homeostasis during the course of infection to influence virus survival, host attachment, and ultimately, disease progression [7].

As reported by Sharif N. et al., a cross-sectional study of 962 participants divided into 8 age groups showed that vitamin D was taken as a supplementation by 55% of positive participants, vitamin C by 55%, calcium by 47%, and zinc by 47%, with effects associated with the presence of symptoms [3]. Furthermore, when supplements were taken as medicine, vitamin C 500 mg once a day, zinc sulfate monohydrate 20 mg single tablet daily, and cholecalciferol (vit D3) 2000 IU single tablet per day, the risk of infection was reduced. However, the possibility of affecting virus replication depends on the duration of supplementation [3].

According to Gualtieri et al. [8], the intake of supplements, such as vitamins A, D, E, K, B6, B12, and C; folates and microelements, such as copper, zinc, selenium, iron, and omega-3 (*n*-3) polyunsaturated fatty acids; and probiotics, such as Lactobacilli and Bifidobacteria, increased host immune defenses and reduced the side effects of vaccination against SARS-CoV-2, especially at the end of the vaccination cycle. These effects were evaluated in 776 Italian subjects, aged between 18 and 86, before and after the vaccination cycle with Comirnaty, Spikevax, Vaxzevria, and Jcovden. Interestingly, a reduction in the side effects of the vaccination, such as fatigue, arthralgia, myalgia, chills, headache, nausea, vomiting, and diarrhea, was observed to have significant correlations with the two sexes, particularly in women taking Vit D and in men after taking prebiotics, *n*-3, and L-glutamine. The effect on symptoms was better after the first vaccination.

During the COVID-19 pandemic, all non-elective surgeries were postponed, including bariatric surgery (BS), leading to a delay in the treatment of obesity and serious consequences [9]. It was demonstrated that hospitalization rates in patients infected with SARS-CoV-2 were lower in severely obese patients undergoing bariatric surgery than in those who did not receive such treatment. The mortality risk decreased from 33 per 1000 in operated patients and 133 per 1000 in non-operated patients [9].

A varied diet rich in vegetables, soy products, nuts, olive oil, fruits and green vegetables, and fatty fish, which is associated with the intake of antioxidant supplements such as coenzyme Q10 (CoQ10) and fiber intake of 27–40 g/daily, exerted specific cardioprotective properties due to the reduction in pro-inflammatory interleukins, platelet aggregation, clot formation, and triglyceride concentration, enhancing the functioning of the blood vessels’ endothelium [10]. In the field of lifestyle medicine, the information collected by Szczepańska E. et al. on nutrients and specific nutritional plans can be useful to all healthcare personnel involved in the prevention, treatment, and reduction of CVD mortality.

Gastrointestinal motor dysfunction, altered intestinal membrane permeability, and visceral hypersensitivity have been recombined in patients with irritable bowel syndrome (IBS) [11]. These symptoms are associated with a broad range of neuropsychiatric abnormalities and psychiatric comorbidities. Gut microbiota-based therapy could be useful to reduce pathogenic microbial populations, such as *Escherichia coli*, *Campylobacter concisus*, *Campylobacter jejuni*, *Helicobacter pylori*, *Clostridium difficile*, *Shigella* spp., and *Salmonella* spp., which are considered a risk factor for the development of functional gastrointestinal disorders in IBS [11]. 

Natural substances from the plant and animal kingdom, which contain principles with anti-inflammatory and immunostimulant activity and are capable of preventing or modulating many viral pathologies, including COVID-19, find application in CCD prevention and therapy, including for neurodegenerative diseases. Multiple sclerosis (MS), characterized by the destruction of myelin due to inflammation and oxidative stress, is related to immune system disorders and neuronal apoptosis [12]. Kheirdeh M. et al. showed that the consumption of royal jelly (RJ), in association with aerobic activity and physical exercise, is effective in reducing the pain threshold in MS as it activates CB1R in the hippocampus thanks to its anti-inflammatory, anti-apoptotic, and antioxidant properties [12].

Considering all of the healthcare costs that each continent faces, it would be crucial to formulate a multidisciplinary strategy to counteract the depletion of an individual’s health status and the predisposition to viral infection and other exogenous risk factors. Therefore, a nutritional strategy to prevent and control CDDs and COVID-19 is urgently needed.
